# Supplementary material for: Uterine cervical neoplasms mass screening at the University Hospital Centre of Libreville, Gabon: Associated factors with precancerous and cancerous lesions
Source: PLoS One. 2021 Jul 23;16(7):e0255289. doi: 10.1371/journal.pone.0255289 (PMC8301670; doi:10.1371/journal.pone.0255289)
Supplement: Attachment — Submitted filename: PONE-D-20-32144_R1.pdf [file pone.0255289.s002.pdf]

PLOS ONE

Cervical cancer screening at the University Hospital Centre of Libreville, Gabon: associated factors with precancerous and cancerous lesions

--Manuscript Draft--

Manuscript Number:	PONE-D-20-32144R1
Article Type:	Research Article
Full Title:	Cervical cancer screening at the University Hospital Centre of Libreville, Gabon: associated factors with precancerous and cancerous lesions
Short Title:	Associated factors with precancerous and cancerous lesions of cervix
Corresponding Author:	SYLVAIN HONORE WOROMOGO, M.D<br>CIESPAC<br>Brazzaville, CONGO
Keywords:	Cervix cancer; screening; risk factors; Neoplasia
Abstract:	The objectives of this study were to identify the associated factors with cancerous and precancerous lesions of cervix. In Africa, the incidence of cervical cancer varies from one region to another, where most women with cervical cancer are seen at an advanced stage. For this reason, cervical cancer screening reduces the incidence and mortality due to this disease, similar to what is being done in Europe. A cross-sectional analytical study was conducted. Socio-demographic characteristics, gynaecological-obstetrical history, risk factors, data from visual inspection with acetic acid and visual inspection with Lugol, colposcopy impressions and results of cytological analysis were performed. A simple and multiple regression were performed to establish a statistically significant difference between certain factors and the presence of precancerous or cancerous lesions of cervix. In this study, of 63 women diagnosed histologically, 43 had precancerous lesions and 20 had cancerous lesions. we found that being older than 35, having the first intercourse before 18, having an antecedent of STI, being a widow and using of tobacco were risk factors associated with precancerous lesions (p = 0.013 with OR=3.44 (1.22-9.73), p = 0.009 with OR = 4.07 (1.69-13.08), p < 0.001 with OR = 3.80 (1.94 -7.47), p < 0.001 with OR = 9.77 (3.87-24.70) and p < 0.001 with OR = 5.47 (2.60-11.52)) respectively. Only being older than 45, being a widow and using tobacco were risk factors associated with cancerous lesions (p = 0.021 with OR = 2.01 (1.58-3.56), p = 0.02 with OR = 2.96 (2.10-3.87), p = 0.041 with OR = 1.98 (1.46-2.44)) respectively. Among participants diagnosed with cervical cancer, there was a significant association with the STI, marital status and smoking. Despite the integration of the detection of precancerous cervical cancer lesions into health facilities in Gabon, cervical cancer ranks second among women's cancers in terms of incidence and first in terms of mortality.
Order of Authors:	SYLVAIN HONORE WOROMOGO, M.D
	Nathalie Ambounda Ledaga
	Felicite Emma Yagata Moussa
	Astride Smeige Mihindou
Response to Reviewers:	Response to Reviewers<br>Reviewer #1<br>Thank you very much for the importance given to our manuscript. We find your comments very pertinent. We have taken these remarks into consideration and corrections have been made in the text.<br>The number of cases of precancerous lesions is higher than that of cancerous lesions for each risk factor studied. This explains why the risk factors are not the same for Tables 4 and 5. We also know that precancerous lesions, if diagnosed in early detection, can be treated and cured.<br>In our database, 8 out of 9 widows with cancerous lesions are over 45 years old. Similarly, all widows with precancerous lesions are over 45 years old. We were therefore unfortunately unable to investigate further to find out the reasons of widowhood.<br>The question of the power of the study is raised as a limitation of the study in the

Powered by Editorial Manager® and ProduXion Manager® from Aries Systems Corporation

	discussion.<br><br>Reviewer #2<br>Thank you very much for the importance given to our manuscript. We find your comments very pertinent. We have taken these remarks into consideration and corrections have been made in the text. Indeed, the objectives of the study were to identify factors associated with cancerous and precancerous cervical lesions. And factors such as age, sexually transmitted infections, smoking and other factors have been incriminated. Nevertheless, we have just included in the discussion the power of the study as a limitation to our study.<br><br>Reviewer #3<br>Thank you very much for the importance given to our manuscript. We find your comments very pertinent. We have taken these remarks into consideration and corrections have been made in the text.<br>We have reviewed the abstract and the references have been removed. The keywords have been revised and extracted from the Mesh controlled vocabulary. The number of the ethical approval protocol for this study is included in the text (methods).<br>We have inserted in the discussion what the research differs from its conventional local treatment. In the "conclusion" section we highlighted how our findings contribute to clinical practice and future research.<br>We have inserted figure 1 for patient inclusion and screening steps. If you think it is not correct, it could be removed from the text.
**Additional Information:**	
**Question**	**Response**
**Financial Disclosure**<br><br>Enter a financial disclosure statement that describes the sources of funding for the work included in this submission. Review the submission guidelines for detailed requirements. View published research articles from *PLOS ONE* for specific examples.<br><br>This statement is required for submission and **will appear in the published article** if the submission is accepted. Please make sure it is accurate.	The authors received no specific funding for this work.

**Unfunded studies**<br>Enter: *The author(s) received no specific funding for this work.*<br><br>**Funded studies**<br>Enter a statement with the following details:<br>• Initials of the authors who received each award<br>• Grant numbers awarded to each author<br>• The full name of each funder<br>• URL of each funder website<br>• Did the sponsors or funders play any role in the study design, data collection and analysis, decision to publish, or preparation of the manuscript?<br>• **NO** - Include this sentence at the end of your statement: *The funders had no role in study design, data collection and analysis, decision to publish, or preparation of the manuscript.*<br>• **YES** - Specify the role(s) played.<br><br>* typeset	
**Competing Interests**<br><br>Use the instructions below to enter a competing interest statement for this submission. On behalf of all authors, disclose any competing interests that could be perceived to bias this work—acknowledging all financial support and any other relevant financial or non-financial competing interests.<br><br>This statement **will appear in the published article** if the submission is accepted. Please make sure it is accurate. View published research articles from *PLOS ONE* for specific examples.	The authors have declared that no competing interests exist.

**NO authors have competing interests**<br><br>Enter: *The authors have declared that no competing interests exist.*<br><br>**Authors with competing interests**<br><br>Enter competing interest details beginning with this statement:<br><br>*I have read the journal's policy and the authors of this manuscript have the following competing interests: [insert competing interests here]*<br><br>* typeset	
**Ethics Statement**<br><br>Enter an ethics statement for this submission. This statement is required if the study involved:<br><br>• Human participants<br>• Human specimens or tissue<br>• Vertebrate animals or cephalopods<br>• Vertebrate embryos or tissues<br>• Field research<br><br>Write "N/A" if the submission does not require an ethics statement.<br><br>General guidance is provided below. Consult the submission guidelines for detailed instructions. **Make sure that all information entered here is included in the Methods section of the manuscript.**	The study was conducted in accordance with the Good Clinical Practices (GCP) guidelines and the regulations of the Ministry of Public Health and Population. The study was approved by the Ethics Committee of the University of Health Sciences, Gabon under the number 007/USS/18.<br>All women admitted to the programme were admitted after obtaining their written informed consent.

Powered by Editorial Manager® and ProduXion Manager® from Aries Systems Corporation

**Format for specific study types**<br><br>**Human Subject Research (involving human participants and/or tissue)**<br>• Give the name of the institutional review board or ethics committee that approved the study<br>• Include the approval number and/or a statement indicating approval of this research<br>• Indicate the form of consent obtained (written/oral) or the reason that consent was not obtained (e.g. the data were analyzed anonymously)<br><br>**Animal Research (involving vertebrate animals, embryos or tissues)**<br>• Provide the name of the Institutional Animal Care and Use Committee (IACUC) or other relevant ethics board that reviewed the study protocol, and indicate whether they approved this research or granted a formal waiver of ethical approval<br>• Include an approval number if one was obtained<br>• If the study involved *non-human primates*, add *additional details* about animal welfare and steps taken to ameliorate suffering<br>• If anesthesia, euthanasia, or any kind of animal sacrifice is part of the study, include briefly which substances and/or methods were applied<br><br>**Field Research**<br><br>Include the following details if this study involves the collection of plant, animal, or other materials from a natural setting:<br>• Field permit number<br>• Name of the institution or relevant body that granted permission	
**Data Availability**<br><br>Authors are required to make all data underlying the findings described fully available, without restriction, and from the time of publication. PLOS allows rare exceptions to address legal and ethical concerns. See the PLOS Data Policy and FAQ for detailed information.	Yes - all data are fully available without restriction

Powered by Editorial Manager® and ProduXion Manager® from Aries Systems Corporation

A Data Availability Statement describing where the data can be found is required at submission. Your answers to this question constitute the Data Availability Statement and **will be published in the article**, if accepted.<br><br>**Important:** Stating 'data available on request from the author' is not sufficient. If your data are only available upon request, select 'No' for the first question and explain your exceptional situation in the text box.<br><br>Do the authors confirm that all data underlying the findings described in their manuscript are fully available without restriction?	
**Describe where the data may be found in full sentences. If you are copying our sample text, replace any instances of XXX with the appropriate details.**<br><br>• If the data are **held or will be held in a public repository**, include URLs, accession numbers or DOIs. If this information will only be available after acceptance, indicate this by ticking the box below. For example: *All XXX files are available from the XXX database (accession number(s) XXX, XXX.)*.<br>• If the data are all contained **within the manuscript and/or Supporting Information files**, enter the following: *All relevant data are within the manuscript and its Supporting Information files.*<br>• If neither of these applies but you are able to provide **details of access elsewhere**, with or without limitations, please do so. For example:<br><br>*Data cannot be shared publicly because of [XXX]. Data are available from the XXX Institutional Data Access / Ethics Committee (contact via XXX) for researchers who meet the criteria for access to confidential data.*<br><br>*The data underlying the results presented in the study are available from (include the name of the third party*	All relevant data are within the manuscript.

and contact information or URL).<br>• This text is appropriate if the data are owned by a third party and authors do not have permission to share the data.<br><br>* typeset	
Additional data availability information:	

Manuscript Click here to access/download;Manuscript;Manuscript.docx

Cervical cancer screening at the University Hospital Centre of Libreville, Gabon : associated factors with precancerous and cancerous lesions

Woromogo Sylvain Honore[1¶*], Ambounda Ledaga Nathalie [1,2 ¶], Yagata-Moussa Felicite Emma[3¶], Mihindou Astride Smeige [2]

[1] InterState Centre for Higher Public Health Education in Central Africa (CIESPAC), Brazzaville, Congo.

[2] University Hospital Centre of Libreville, Gynecology and Obstetric Service, Gabon.

[3]Faculty of Health Sciences, University of Bangui, Central African Republic

***Corresponding author :**

Sylvain Honore Woromogo; MD; MPH - Epidemiology

Inter-States Center of Higher Education in Public Health of Central Africa, Brazzaville, Congo

Email: woromogos@gmail.com

Tel : 00242 05 090 48 12

Abstract

The objectives of this study were to identify the associated factors with cancerous and precancerous lesions of cervix. In Africa, the incidence of cervical cancer varies from one region to another, where most women with cervical cancer are seen at an advanced stage. For this reason, cervical cancer screening reduces the incidence and mortality due to this disease, similar to what is being done in Europe. A cross-sectional analytical study was conducted. Socio-demographic characteristics, gynaecological-obstetrical history, risk factors, data from visual inspection with acetic acid and visual inspection with Lugol, colposcopy impressions and results of cytological analysis were performed. A simple and multiple regression were performed to establish a statistically significant difference between certain factors and the presence of precancerous or cancerous lesions of cervix. In this study, of 63 women diagnosed histologically, 43 had precancerous lesions and 20 had cancerous lesions. we found that being older than 35, having the first intercourse before 18, having an antecedent of STI, being a widow and using of tobacco were risk factors associated with precancerous lesions ($p = 0.013$ with OR=3.44 (1.22-9.73), $p = 0.009$ with OR = 4.07 (1.69-13.08), $p < 0.001$ with OR = 3.80 (1.94 -7.47), $p < 0.001$ with OR = 9.77 (3.87-24.70) and $p < 0.001$ with OR = 5.47 (2.60-11.52)) respectively. Only being older than 45, being a widow and using tobacco were risk factors associated with cancerous lesions ($p = 0.021$ with OR = 2.01 (1.58-3.56), $p = 0.02$ with OR = 2.96 (2.10-3.87), $p = 0.041$ with OR = 1.98 (1.46-2.44)) respectively. Among participants diagnosed with cervical cancer, there was a significant association with the STI, marital status and smoking. Despite the integration of the detection of precancerous cervical cancer lesions into health facilities in Gabon, cervical cancer ranks second among women's cancers in terms of incidence and first in terms of mortality.

Key words : Cervix cancer. Screening. Risk factors. Neoplasia.

Introduction

Cervical cancer is caused by untreated and undiagnosed precancerous lesions that are not diagnosed in time. According to GLOBOCAN 2018, the number of new cases of cervical cancer is estimated at 569,847 representing 3.2% of all 36 cancers with 569,847 deaths representing 3.2% of all cancer deaths[1]. Also, standardized incidence rates per 100,000 and standardized mortality rates per 100,000 were higher in developing countries than in developed countries for cervical cancer with eight versus five and four versus two respectively[2]. Each year more than 500 women are diagnosed with uterine cancer and the disease causes more than 300,000 deaths worldwide[3]. In Africa, the incidence of cervical cancer varies from one region to another, particularly in developing countries where most women with cervical cancer are seen at an advanced and often incurable stage or are only suitable for palliation[4,5]. For this reason, cervical cancer screening reduces the incidence and mortality due to this disease[6], similar to what is being done in Europe[7]. The World Health Organization (WHO) and other agencies have proposed guidelines for screening[6,8,9] and recommendations for abnormal cervical smears[10-12]. In Gabon, early detection of cervical cancer has been integrated into health facilities since 2014. But little data is available on the prevalence of precancerous lesions in diagnostic centres. The objective of this study is to determine the prevalence of precancerous cervical lesions in women screened at University Hospital Centre of Libreville (UHCL) in 2018 and to identify the factors associated with these lesions.

Materials and Methods

Type of study

This was a cross-sectional analytical study conducted during the period from January 1, 2018 to December 31, 2018. Women who came for screening and agreed to participate in the study were consecutively included.

Study population

The study population consisted of women aged 25 to up 65 years old who were screened for cervical cancer in Libreville. Sensitization on cervical cancer screening was done by radio advertisements and with the help of community health workers before and during the screening period. Participants were recruited from the population of Libreville without regard to HIV status. Women were excluded from the study if they were more than 20 weeks pregnant; less than 12 weeks post-partum; had a previous history of treatment of cancerous lesions; had a known allergy to acetic acid; or if they had undergone total hysterectomy.

Data collection procedures

Screening was done by medical doctors, nurses, and midwives with 3 years of experience in maternity service who had been trained for 2 weeks in theory and practice in cervical cancer screening. The data were collected from a questionnaire and results forms for cytopathological anatomy analysis. This screening consisted in carrying out successively (Figure 1):

- A speculum examination during which the Visual inspection after application of acetic acid (VIA) and lugol's iodine (VIL) tests are performed for screening. When there is an anomaly in the VIA and VIL (positive VIA, VIL test), a colposcope examination is systematically

performed to clarify the lesion and guide the biopsy. The biopsy fragments are immediately attached to 10% formaldehyde and sent to the laboratory for histological examination.

The histological results of the piece are brought back to the centre by the participant herself in order to develop a therapeutic plan and follow-up with a well-defined monitoring rhythm and consequent intervention.

All women were treated according to the protocol recommended by National Agency for Health Accreditation and Evaluation[11,13].

Variables

The following variables were studied: socio-demographic characteristics, gynaecological-obstetrical history, risk factors, data from visual inspection with acetic acid and visual inspection with Lugol, colposcopy impressions and results of cytological and histological analysis when a biopsy was performed. Two dependent variables of interest were chosen. Invasive cervical cancer was defined as a binary variable (yes/no) with an outcome of diagnosis of invasive cervical cancer. Invasive cervical cancer lesions were diagnosed clinically if lesions suggestive of invasive cancer were found on the cervix. All cases were confirmed by colposcopy and biopsy. Cervical pre-cancer was defined as a binary variable. Cervical pre-cancer lesions were diagnosed clinically after application of acetic acid. Independent variables included socio-demographic variables : these included age, sex, educational level, marital status, tobacco, age of first intercourse, STIs, hormonal status, gesture, parity.

Statistical analysis

Text and table entry was done on Word and Excel 2016 software and data analysis was done with EpiInfo 7.2 softwares. The socio-demographic and gynaecological characteristics as well as the results of various tests were obtained by simple frequencies. Summary statistics such as

mean and standard deviation were calculated for continuous variables. Bivariate methods were used to show the relationship between variables ; contingency tables were used to describe relationship between categorical variables. Tabular methods of describing the relationship between two nominal variables by finding proportions were also employed. Multivariate analysis was used to determine the socio-demographic, behavioral and clinical factors associated with cervical cancer and pre-cancer lesions. Binary and multivariate logistic regressions were used to show the association of any cervical cancerous lesion with different factors To facilitate regressions, most variables were made into binary variables (age group, marital status, education level, social economic status, number of pregnancies, number of children born, age of the first intercourse) to facilitate analysis of the association of cervical cancer and precancer with different factors.

The Chi-square and Wald tests were used as well as the odds ratio with their 95% confidence interval at the 5% threshold. Multiple logistic regression was used to establish the relationship between socio demographic and gynecological characteristics and precancerous or cancerous lesions. Multivariable logistic regression analyses with backward elimination stepwise selection with $p < 0.20$ were used to identify baseline explication that predicts precancerours or cancerous lesions.

Ethical considerations

The study was conducted in accordance with the Good Clinical Practices (GCP) guidelines and the regulations of the Ministry of Public Health and Population. The study was approved by the Ethics Committee of the University of Health Sciences, Gabon under the number 007/USS/18. All women admitted to the programme were admitted after obtaining their written informed consent.

Results

The average age of the women screened was 39.8 ± (SD =11.4) years with extremes of 25 and 83 years. Women aged 25-34 were the most represented, in the order of 40.5% of the sample. There were 625 women with a history of sexually transmitted infections, 41.3%. Of these, 534 had Chlamydia infection, 35 had HIV and 56 had gonorrhoea and syphilis. Smoking women accounted for 5.8% of the participants. Among the participants screened, 67.8% did not use a contraceptive method. The other characteristics of women are presented in Table 1.

Visual inspection and colposcopy results

In this study, 12.0% of women had a pathological cervix on unprepared examination. Women who tested positive for VIA and VIL accounted for 5.9% and 12.6% respectively. Colposcopy was only for cases with positive VIL, 191 tests or 12.6% (191/1512). With colposcopy, 46.1% of women had atypical grade1 transformations, 49.7% grade 2 and 4.2% had both atypical transformations (Table 2).

Histological results

In this study, of 63 women diagnosed histologically, 43 had precancerous lesions with 23 CIN I or 36.5%; 11 CIN II or 17.5% and 9 CIN III or 14.3%. Squamous cell cancers accounted for 20.6% and adenocarcinomas 3.2% (Table 3).

Risk factors of precancerous and cancerous lesions

Tables 4 and 5 show the characteristics that were risk factors for precancerous and cancerous lesions using bivariate and multivariable analysis. Using binary logistic regression to assess the relationship between cervical precancerous lesions and the independent variables collected, we found that being older than 35, having the first intercourse before 18, having an antecedent of

STI, being a widow and use of tobacco were risk factors associated with precancerous lesions (p = 0.013 with OR=3.44 (1.22-9.73), p = 0.009 with OR = 4.07 (1.69-13.08), p < 0.001 with OR = 3.80 (1.94 -7.47), p < 0.001 with OR = 9.77 (3.87-24.70) and p < 0.001 with OR = 5.47 (2.60-11.52)) respectively. With multivariate regression, the risk factors of developing any precancerous lesion were the same but the OR decreased with increased parity (p = 0.04 with OR=1.44 (1.09-2.21), p = 0.04 with OR = 2.01 (1.99-2.61), p = 0.006 with OR = 1.97 (1.68-2.89), p = 0.021 with OR = 1.99 (1.30-2.37) and p = 0.02 with OR = 2.29 (1.14-2.99)) respectively. The total number of pregnancies and parity were not associated with precancerous lesions and cervical cancer. But after adjustement, only being older than 45, being a widow and using tobacco were risk factors associated with cancerous lesions (p = 0.021 with OR = 2.01 (1.58-3.56), p = 0.02 with OR = 2.96 (2.10-3.87), p = 0.041 with OR = 1.98 (1.46-2.44)) respectively.

Discussion

This study provided an update on the prevalence of precancerous cervical lesions in the obstetrics and gynaecology department. The target population was sexually active women 25 years of age and older. Several countries have succeeded in preventing 91% of all invasive cervical cancers by implementing generalized cytological screening[14]. Finally, the authors could have screened as many women in order to increase the power of the study.

Socio-demographic and gyneco-obstetrical characteristics

In this study, the mean age was 39.8 ±11.4 years with extremes ranging from 25 to 83 years. Some authors have worked on the same subject as us. For these authors, the average age ranged from 33 - 40 years[7,9,10,14-17] and was close to ours. The age at which screening begins is

the subject of much debate among cervical cancer screening professionals. At the global level, there is no consensus on the age at which screening should begin. At 21 years of age (or one year after the first reports) in the United States and Switzerland[2], 25 years in England, France and according to WHO[3,4]. Cervical cancer is caused by an oncogenic sexually transmitted HPV infection. Therefore, any woman who has already had sexual intercourse should be regularly screened. The majority of the study population were single and concubine women (67.7%), however 28.1% were married. These results are similar to those found by MPIGA et *al*[15] . The age at which screening begins in Gabon is 25 years old. It was found that 80.4 % of participants wre premenopausal. Paradoxically, 67.8% of women were not using contraceptives. This translates into a small percentage of nulliparous people. In Gabon, women have the perception that contraception makes them sterile.

Screening results

In this series, 91 women had a pathological cervix, or 2%. The VIA and VIL tests were positive at 5.9% and 12.6% respectively. These results vary in some authors from 1.9% to 10.8% for VIA[10,15,18-20] and from 4.8% to 5.8% for VIL[13,15]. This difference is explained by the fact that women have been sensitized with an important message : cervical cancer screening reduces the risk of disease and mortality. The cytohistological analysis determined the prevalence of precancerous lesions at 2.8%. This prevalence seems high because of the large number of women screened, compared to some authors[20]. The screening programme should focus on raising awareness among women, which is generally very successful and has shown that countries that have implemented cervical cancer screening reduce the burden of this disease[21,22].

Risk factors

We found that 41.3% of the women screened reported having contracted a STI. In our data base most of them contracted chlamydia infection and gonococcal disease. This high percentage of chlamydia infection is of concern ; Bahmanyar et *al* in 2012 has established that history of sexually transmitted infections (STIs) is significantly associated with a risk of cervical injury[23]. Unprotected sexual activity is associated with an increased risk of contracting STIs, leading to cervical inflammation which can promote the development of cervical dysplasia [24]. In this study 5.8% of the participants were smokers. Smoking seems to be strongly associated with the development of precancerous cervical lesions and cancer. Smoking is one of the most commonly identified environmental co-factors that can affect the risk of cervical cancer. The majority of women screened, 53.1% had their first sexual intercourse between 12-17 years of age. This result marks the precocity of sexual intercourse, which is a factor that favours precancerous lesions of the cervix[15,17,25]. It is important to continue with raising awareness among women about screening. Compared to married women, widows are more prone to develop precancerous and cancerous cervical lesions. We could not explore the reasons for widowhood, but many of these widows are on antiretroviral treatment. These results confirm the interest of organized cervical cancer screening in Gabon. The countries that have implemented this screening programme have made a significant contribution to reducing the burden of this disease[22].

This study had several limitations. As the study was a convenience sample of women presenting for care from only Libreville in Gabon, the prevalence results may not be generalizable to the whole of Gabon or to the Central African female population, where HIV infection rates are quite varied. There is also a possibility of selection bias, as women who participated in the screening program may have been more likely to have current symptoms. The main purpose of

this study was to serve as a baseline for further studies and to help in planning for cervical cancer screening in Gabon ; future studies using randomized sampling techniques may give a more complete estimation of the national prevalence. Lastly, there were limited variables available for analysis in the medical records, limiting the number of risk factors and confounders that we were able to study, for exemple the antecedent of cancer family.

Conclusion

In this study, the participants in the cervical cancer screening at the UHCL in 2018 had an average age of 39 years, they were mostly single, premenopausal, multi-gestured. Early sexual intercourse and smokers were found. Among participants diagnosed with cervical cancer, there was a significant association with age, STI, marital status and smoking. Despite the integration of the detection of precancerous cervical cancer lesions into health facilities in Gabon, cervical cancer ranks second among women's cancers in terms of incidence and first in terms of mortality. These results help to further strengthen the fight against cervical cancer, provide clinicians with the characteristics of women with cervical cancer and allow further research to identify the factors that contribute to it. Thus, the detection and management of precancerous cervical lesions could reduce the mortality rate from cervical cancer in the long term.

Fundings : None

Conflicts of interest : The authors declare no conflict of interest

Authors' contributions :

NAL designed the study, wrote the protocol and text and supervised the data analysis. SHW, ASM and FEYM participated in the drafting of the protocol, data analysis and text writing. All authors have read and approved the manuscript and contributed equally.

Acknowledgements :

The authors would like to thank the Ministry of Health of Gabon

Declarations : Availability of data and materials

The datasets used and analysed during the current study available from the corresponding author on reasonable request. The datasets generated and/or analysed during the current study are not publicly available due to the promise made to women to keep the data confidential when they are questioned, but are available from the corresponding author on reasonable request.

References

1. Bray F, Ferlay J, Soerjomataram I, Siegel RL, Torre LA, Jemal A. Global cancer statistics 2018: GLOBOCAN estimates of incidence and mortality worldwide for 36 cancers in 185 countries. CA Cancer J Clin 2018 ; 68 : 394-424 doi: 10.3322/caac.21492
2. Fitzmaurice C et al. The Global Burden of Cancer 2013. JAMA Oncol 2015 ; 1 : 505-27 doi: 10.1001/jamaoncol.2015.0735
3. Cohen PA, Jhingran A, Oaknin A, Denny L. Cervical Cancer. Lancet 2019 ; 3 : 169-182 doi: 10.1016/S0140-6736(18)32470-X.
4. Denny L, Anorlu R. Cervical cancer in Africa. Cancer Epidemiol Biomarkers Prev 2012 ; 21 : 1434-8 DOI: 10.1158/1055-9965.EPI-12-0334
5. Denny L. Cervical cancer :prevention and treatment. Discov Med 2012 ; 14 : 125-31
6. Comprehensive cervical cancer control : a guide to essential practice – 2nd ed. Geneva : World Health Organization ; 2017. Licence : CC BY-NC-SA 3.0 IGO

7. Denny L, Kuhn L, Pollack A, Wright TC Jr. Direct visual inspection for cervical cancer screening : an analysis of factors influencing test performance. Cancer 2002 ; 94 : 1699-707 DOI :10.1002/cncr.10381
8. Elfström KM, Arnheim-Dahlström L, von Karsa L, Dillner J. Cervical cancer screening in Europe : Quality assurance and organisation of programmes. Eur J Cancer 2015 ; 51 : 950-68
9. Saslow D, Solomon D, Lawson HW, Killackey M, Kulasingam SL, Cain J *et al*. American Cancer Society, American Society for Colposcopy and Cervical Pathology, and American Society for Clinical Pathology screening guidelines for the prevention and early detection of cervical cancer. CA Cancer J Clin 2012 ; 62 : 147-72
10. Sandjong Tietchou I, Sando Z, Tebeu PM, Mouelle Sone A, Essame Oyono JL, Doh Anderson S. Evaluation of the prevention and treatment of the precancerous lesions of the uterine cervix according to the 'see and treat' approach. Health Sci Dis 2015 ; 16
11. National Agency for Health Accreditation and Evaluation. Conduct in presence of an abnormal cervical smear. J Gynecol Obstet Biol Reprod 1999 ; 28 :310-18
12. Hasanzadeh M, Esmaeili H, Tabaee S, Samadi F. Evaluation of visual inspection with acetic acid as a feasible screening test for cervical neoplasia. J Obstet Gynaecol Res 2011 ; 37 :1802-6 doi: 10.1111/j.1447-0756.2011.01614.x.
13. Santesso N, Mustafa RA, Schünemann HJ, Arbyn M, Blumenthal PD, Cain J *et al*. World Health Organization Guidelines for treatment of cervical intraepithelial neoplasia 2-3 and screen-and-treat strategies to prevent cervical cancer. Int J Gynaecol Obstet 2016 ; 132 : 252-8 doi: 10.1016/j.ijgo.2015.07.038
14. Denny L, Herrero R, Levin C *et al*. Cervical Cancer. In: Gelband H, Jha P, Sankaranarayanan R, et al., editors. Cancer : Disease Control Priorities, Third Edition (Volume 3). Washington (DC): The International Bank for Reconstruction and

Development / The World Bank; 2015 Nov 1. Chapter 4. Available from: https://www.ncbi.nlm.nih.gov/books/NBK343648/ doi: 10.1596/978-1-4648-0349-9_ch4

15. Mpiga E, Ivanga M, Koumakpayi IH, Engohan-Aloghe C, Ankély JC, Belembaogo E *et al*. Interest in visual inspection with acetic acid and Lugol iodine with colposcope in screening of cervical lesions in Gabon. Pan Afr Med J 2015 ; 22 : 165 doi: 10.11604/pamj.2015.22.165.7038

16. Millogo FT, Akotionga M, Lankoande J. Cervix cancer screening in a health district (Burkina Faso) by biopsies after the application of acetic acid and lugol. Bull Soc Pathol Exot 2004 ; 97 : 135-38

17. Salifou K, Brun L, Akpona LFJ, Obossou AAA, Perrin RX. Factors associated with precancerous and cancerous cervical lesions in the city of Parakou, Benin. Eur Sci J 2015 ; 11 : 275-83

18. Phongsavan K, Phengsavanh A, Wahlström R, Marions L. Safety, feasibility, and acceptability of visual inspection with acetic acid and immediate treatment with cryotherapy in rural Laos. Int J Gynaecol Obstet 2011 ; 114 :268-72 doi: 10.1016/j.ijgo.2011.03.009.

19. Fallala MS, Mash R. Cervical cancer screening : Safety, acceptability, and feasibility of a single-visit approach in Bulawayo, Zimbabwe. Afr J Prim Health Care Fam Med 2015 ; 7 : doi: 10.4102/phcfm.v7i1.742.

20. Ngoma T, Muwonge R, Mwaiselage J, Kawegere J, Bukori P, Sankaranarayanan R. Evaluation of cervical visual inspection screening in Dar es Salaam, Tanzania. Int J Gynaecol Obstet 2010 ; 109 : 100-4 doi: 10.1016/j.ijgo.2009.11.025

21. Adefuye PO, Dada OA, Adefuye BO, Shorunmu TO, Akinyemi BO, Idowu-Ajiboye BO. Feasibility, acceptability, and effectiveness of visual inspection of the cervix with acetic

acid and cryotherapy for dysplasia in Nigeria. Int J Gynaecol Obstet 2015 ; 129 : 62-6 doi: 10.1016/j.ijgo.2014.10.032

22. Denny L, Anorlu R. Cervical cancer in Africa. Cancer Epidemiol Biomarkers Prev 2012 ; 21 : 1434-8 DOI: 10.1158/1055-9965.EPI-12-0334
23. Roset Bahmanyar E, Paavonen J, Naud P, Salmerón J, Chow SN, Apter D *et al*. Prevalence and risk factors for cervical HPV infection and abnormalities in young adult women at enrolment in the multinational PATRICIA trial. Gynecol Oncol 2012 ; 127 :440-50 doi: 10.1016/j.ygyno.2012.08.033
24. Adefuye A, Sales K. Regulation of inflammatory pathways in cancer and infectious disease of the cervix. Scientifica (Cairo) 2012 ; 2012: 548150.PubMed/Google scholar
25. Makuza JD, Nsanzimana S, Muhimpundu MA, Pace LE, Ntaganira J, Riedel JR. Prevalence and risk factors for cervical cancer and precancerous lesions in Rwanda. Pan Afr Med J 2015 ; 22 : 26 doi:10.11604/pamj.2015.22.26.7116

Table 1. Sociodemographic and medical characteristics of participants

Variables		Number	Percentage
Age (years)			
	25-34	613	40.5
	35-44	427	28.2
	45-54	292	19.3
	55-64	141	9.3
	65 +	39	2.6
Marital status			
	Married	425	28.1
	Concubine/single	1024	67.7
	Widow	63	4.2
Hormonal status			
	Postmenopausal	297	19.6
	Premenopausal	1215	80.4
Current contraceptive use			
	Yes	487	32.2
	No	1025	67.8
Gesture			
	Nulligest	155	10.3
	Primigest	154	10.2
	Multigest	1203	79.5
Parity			
	Nulliparous	206	13.6
	Primiparous	308	20·4
	Multiparous	998	66·0
First intercourse (years)			
	< 12	41	2.7
	12-17	802	53.1
	18-29	669	44.2
STIs			
	Yes	625	41.3
	No	887	58.7
Tobacco			
	Yes	87	5.8
	No	1425	94.2

Table 2. Distribution of women according to different types of tests

Visual inspection	N		Number	Percentage
Unprepared test	1512			
		Normal	1327	88.0
		Pathological	185	12.0
VIA test	1512			
		Negative	1423	94.1
		Positive	89	5.9
VIL test	1512			
		Negative	1321	87.4
		Positive	191	12.6
Colposcopy*	191			
		ATG 1	88	46.1
		ATG 2	95	49.7
		ATG 1 & ATG 2	08	4.2

* Positive cases only from VIL test

Table 3. Results from histological examinations for screened women

	Histology	Number	Percentage
Precancerous lesions			
	CIN 1	23	36.5
	CIN 2	11	17.5
	CIN 3	9	14.3
Cervical cancers			
	Squamous cell cancers	13	20.6
	Adenocarcinomas	07	11.1
Total		63	100.0

Table 4. Risk factors of precancerous lesions

Risk factors	Histology					
	Injury	No injury	OR (95% CI)	p	AOR* (95% CI)	p
Age (years)						
25-34	05	608		1.0	1.0	-
35-44	25	402	7.56 (2.87-19.91)	< 0.001	2.81 (2.09-3.11)	0.05
45 +	13	459	3.44 (1.22-9.73)	0.013	1.44 (1.09-2.21)	0.04
Age on first intercourse						
< 12	05	36	6.06 (2.08-17.59)	0.004	1.66 (1.11 – 3.36)	0.03
12-17	23	779	4.07 (1.69-13.08)	0.009	2.01 (1.99-2.61)	0.04
18-29	15	654	1.0	-	1.0	
STIs						
Yes	31	594	3.80 (1.94 -7.47)	< 0.001	1.97 (1.68-2.89)	0.006
No	12	875	1.0	-	1.0	-
Marital status						
Married	09	416	1.0	-		
Concubine/Single	23	1021	1.04 (0.48-2.27)	0.55		
Widow	11	52	9.77 (3.87-24.70)	< 0.001	1.99 (1.30-2.37)	0.021
Tobacco						
Yes	10	77	5.47 (2.60-11.52)	< 0.001	2.29 (1.14-2.99)	0.02
No	33	1392	1.0		1.0	

* : Adjusted Odds ratio. Adjustment on gesture, parity and use of oral contraceptive

Table 5 : Risk factors for cancerous lesions

Risk factors	Histology					
	Injury	No injury	OR (95% CI)	p	AOR* (95% CI)	p
Age (years)						
25-34	3	610	1.0		1.0	
35-44	6	421	2.89 (0.72-11.65)	0.11	-	
45 +	11	461	4.85 (1.35-17.49)	0.008	2.01 (1.58-3.56)	0.021
Age on first intercourse						
< 12	4	37	10.22 (2.86-36.49)	0.0023	1.88 (0.97 – 2.01)	0.087
12-17	9	793	1.17 (0.40-2.89)	0.547		
18-29	7	662	1.0	-		
STIs						
Yes	14	611	3.36 (1.29 -8.80)	0.008	1.97 (1.68-2.89)	0.050
No	06	881	1.0	-	1.0	-
Marital status						
Married	04	421	1.0	-		
Concubine/Single	07	1017	0.72 (0.21-2.49)	0.410		
Widow	09	63	15.03 (4.49-50.28)	< 0.001	2.96 (2.10-3.87)	0.025
Tobacco						
Yes	05	82	5.73 (2.03-16.16)	0.004	1.98 (1.46-2.44)	0.041
No	15	1410	1.0		1.0	

* : Adjusted Odds ratio. Adjustment on gesture, parity and use of oral contraceptive

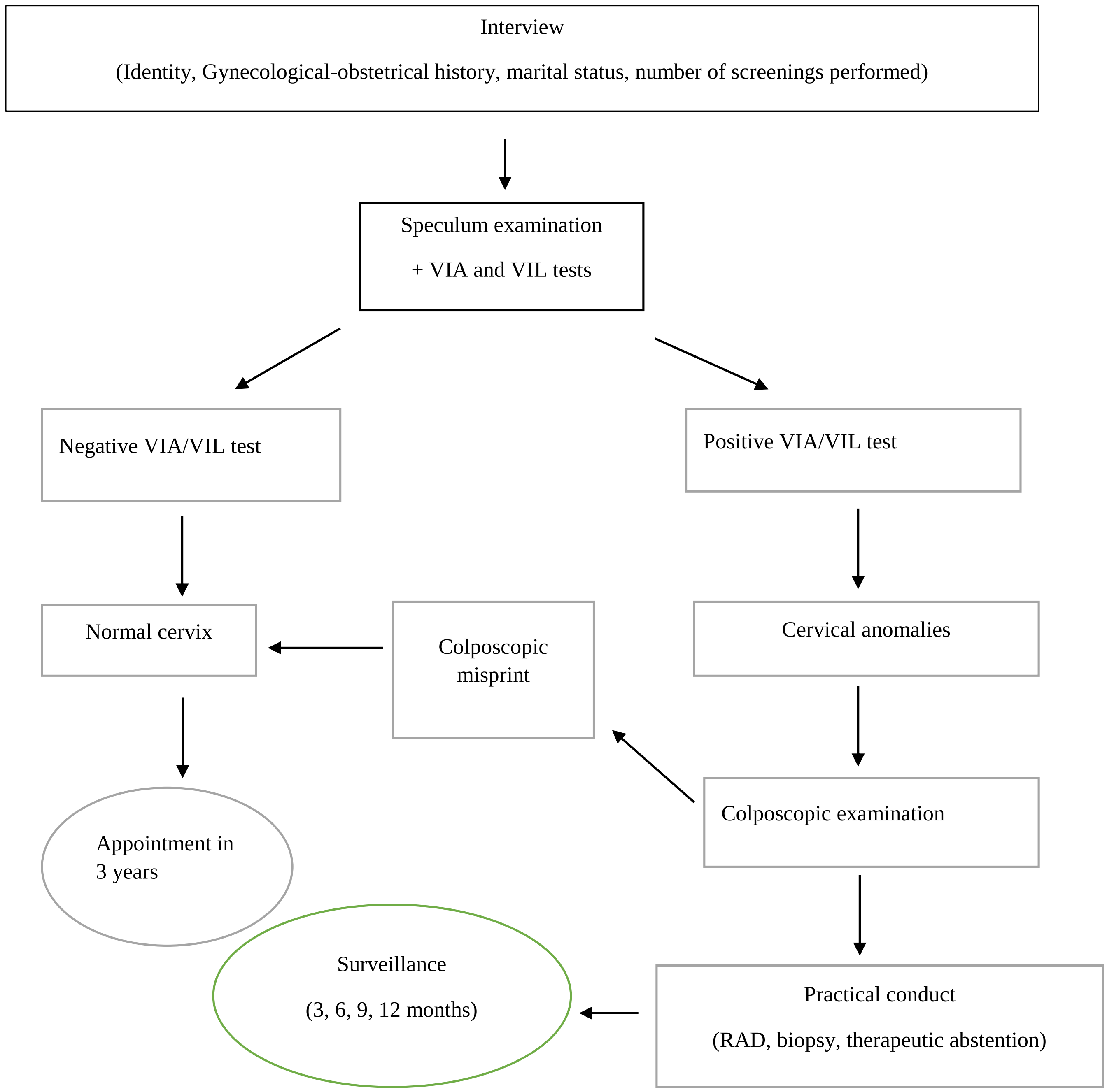

Figure 1. Patient inclusion and screening steps

Cervical cancer screening at the University Hospital Centre of Libreville, Gabon : associated factors with precancerous and cancerous lesions

Woromogo Sylvain Honore[1¶*], Ambounda Ledaga Nathalie [1,2 ¶], Yagata-Moussa Felicite Emma[3¶], Mihindou Astride Smeige [2]

[1] InterState Centre for Higher Public Health Education in Central Africa (CIESPAC), Brazzaville, Congo.

[2] University Hospital Centre of Libreville, Gynecology and Obstetric Service, Gabon.

[3]Faculty of Health Sciences, University of Bangui, Central African Republic

***Corresponding author :**

Sylvain Honore Woromogo; MD; MPH - Epidemiology

Inter-States Center of Higher Education in Public Health of Central Africa, Brazzaville, Congo

Email: woromogos@gmail.com

Tel : 00242 05 090 48 12

Abstract

The objectives of this study were to identify the associated factors with cancerous and precancerous lesions of cervix. According to GLOBOCAN 2018, the number of new cases of cervical cancer is estimated at 569,847 representing 3.2% of all 36 cancers. In Africa, the incidence of cervical cancer varies from one region to another, where most women with cervical cancer are seen at an advanced stage. For this reason, cervical cancer screening reduces the incidence and mortality due to this disease, similar to what is being done in Europe. A cross-sectional analytical study was conducted. Socio-demographic characteristics, gynaecological-obstetrical history, risk factors, data from visual inspection with acetic acid and visual inspection with Lugol, colposcopy impressions and results of cytological analysis was were performed. A simple and multiple regression were performed to establish a statistically significant difference between certain factors and the presence of precancerous or cancerous lesions of cervix. In this study, of 63 women diagnosed histologically, 43 had precancerous lesions and 20 had cancerous lesions. we found that being older than 35, having the first intercourse before 18, having an antecedent of STI, being a widow and using of tobacco were risk factors associated with precancerous lesions ($p = 0.013$ with OR=3.44 (1.22-9.73), $p = 0.009$ with OR = 4.07 (1.69-13.08), $p < 0.001$ with OR = 3.80 (1.94 -7.47), $p < 0.001$ with OR = 9.77 (3.87-24.70) and $p < 0.001$ with OR = 5.47 (2.60-11.52)) respectively. Only being older than 45, being a widow and using tobacco were risk factors associated with cancerous lesions ($p = 0.021$ with OR = 2.01 (1.58-3.56), $p = 0.02$ with OR = 2.96 (2.10-3.87), $p = 0.041$ with OR = 1.98 (1.46-2.44)) respectively. Among participants diagnosed with cervical cancer, there was a significant association with the STI, marital status and smoking. Despite the integration of the detection of precancerous cervical cancer lesions into health facilities in Gabon, cervical cancer ranks second among women's cancers in terms of incidence and first in terms of mortality.

Key words : ~~Cervical~~ Cervix cancer. Screening. Risk ~~Associated~~ factors. ~~Cancerous lesions~~Neoplasia. ~~Precancerous lesions~~

Introduction

Cervical cancer is caused by untreated and undiagnosed precancerous lesions that are not diagnosed in time. According to GLOBOCAN 2018, the number of new cases of cervical cancer is estimated at 569,847 representing 3.2% of all 36 cancers with 569,847 deaths representing 3.2% of all cancer deaths[1]. Also, standardized incidence rates per 100,000 and standardized mortality rates per 100,000 were higher in developing countries than in developed countries for cervical cancer with eight versus five and four versus two respectively[2]. Each year more than 500 women are diagnosed with uterine cancer and the disease causes more than 300,000 deaths worldwide[3]. In Africa, the incidence of cervical cancer varies from one region to another, particularly in developing countries where most women with cervical cancer are seen at an advanced and often incurable stage or are only suitable for palliation[4,5]. For this reason, cervical cancer screening reduces the incidence and mortality due to this disease[6], similar to what is being done in Europe[7]. The World Health Organization (WHO) and other agencies have proposed guidelines for screening[6,8,9] and recommendations for abnormal cervical smears[10-12]. In Gabon, early detection of cervical cancer has been integrated into health facilities since 2014. But little data is available on the prevalence of precancerous lesions in diagnostic centres. The objective of this study is to determine the prevalence of precancerous cervical lesions in women screened at University Hospital Centre of Libreville (UHCL) in 2018 and to identify the factors associated with these lesions.

Materials and Methods

Type of study

This was a cross-sectional analytical study conducted during the period from January 1, 2018 to December 31, 2018. Women who came for screening and agreed to participate in the study were consecutively included.

Study population

The study population consisted of women aged 25 to up 65 years old who were screened for cervical cancer in Libreville. Sensitization on cervical cancer screening was done by radio advertisements and with the help of community health workers before and during the screening period. Participants were recruited from the population of Libreville without regard to HIV status. Women were excluded from the study if they were more than 20 weeks pregnant; less than 12 weeks post-partum; had a previous history of treatment of cancerous lesions; had a known allergy to acetic acid; or if they had undergone total hysterectomy.

Data collection procedures

Screening was done by medical doctors, nurses, and midwives with 3 years of experience in maternity service who had been trained for 2 weeks in theory and practice in cervical cancer screening. The data were collected from a questionnaire and results forms for cytopathological anatomy analysis. This screening consisted in carrying out successively:

- A speculum examination during which the Visual inspection after application of acetic acid (VIA) and lugol's iodine (VIL) tests are performed for screening. When there is an anomaly in the VIA and VIL (positive VIA, VIL test), a colposcope examination is systematically

performed to clarify the lesion and guide the biopsy. The biopsy fragments are immediately attached to 10% formaldehyde and sent to the laboratory for histological examination.
The histological results of the piece are brought back to the centre by the participant herself in order to develop a therapeutic plan and follow-up with a well-defined monitoring rhythm and consequent intervention.

All women were treated according to the protocol recommended by National Agency for Health Accreditation and Evaluation[11,13].

Variables

The following variables were studied: socio-demographic characteristics, gynaecological-obstetrical history, risk factors, data from visual inspection with acetic acid and visual inspection with Lugol, colposcopy impressions and results of cytological and histological analysis when a biopsy was performed. Two dependent variables of interest were chosen. Invasive cervical cancer was defined as a binary variable (yes/no) with an outcome of diagnosis of invasive cervical cancer. Invasive cervical cancer lesions were diagnosed clinically if lesions suggestive of invasive cancer were found on the cervix. All cases were confirmed by colposcopy and biopsy. Cervical pre-cancer was defined as a binary variable. Cervical pre-cancer lesions were diagnosed clinically after application of acetic acid. Independent variables included socio-demographic variables : these included age, sex, educational level, marital status, tobacco, age of first intercourse, STIs, hormonal status, gesture, parity.

Statistical analysis

Text and table entry was done on Word and Excel 2016 software and data analysis was done with EpiInfo 7.2 softwares. The socio-demographic and gynaecological characteristics as well as the results of various tests were obtained by simple frequencies. Summary statistics such as

mean and standard deviation were calculated for continuous variables. Bivariate methods were used to show the relationship between variables ; contingency tables were used to describe relationship between categorical variables. Tabular methods of describing the relationship between two nominal variables by finding proportions were also employed. Multivariate analysis was used to determine the socio-demographic, behavioral and clinical factors associated with cervical cancer and pre-cancer lesions. Binary and multivariate logistic regressions were used to show the association of any cervical cancerous lesion with different factors To facilitate regressions, most variables were made into binary variables (age group, marital status, education level, social economic status, number of pregnancies, number of children born, age of the first intercourse) to facilitate analysis of the association of cervical cancer and precancer with different factors. ~~After bivariate analysis, a series of multivariate regressions were conducted: all variables with P-value below 0.10 in bivariate analyses were included in multivariate regression, after those variables with p-value <0.10 with cofounding variables were also included in another series of multivariate regression to find out final variables associated with any cervical cancerous lesion and precancerous lesions.~~

The Chi-square and Wald tests were used as well as the odds ratio with their 95% confidence interval at the 5% threshold. Multiple logistic regression was used to establish the relationship between socio demographic and gynecological characteristics and precancerous or cancerous lesions. Multivariable logistic regression analyses with backward elimination stepwise selection with $p < 0.20$ were used to identify baseline explication that predicts precancerours or cancerous lesions.

Ethical considerations

The study was conducted in accordance with the Good Clinical Practices (GCP) guidelines and the regulations of the Ministry of Public Health and Population. The study was approved by the

Formatted: Font: (Default) Times New Roman, 12 pt

Formatted: Justified, Line spacing: Double

Formatted: Font: (Default) Times New Roman, 12 pt

Formatted: Font: (Default) Times New Roman, 12 pt

Formatted: Font: (Default) Times New Roman, 12 pt

Formatted: Font: (Default) Times New Roman, 12 pt

Formatted: Font: (Default) Times New Roman, 12 pt

Formatted: Font: (Default) Times New Roman, 12 pt, Not Italic

Formatted: Font: (Default) Times New Roman, 12 pt

Formatted: Font: (Default) Times New Roman, 12 pt, Not Italic

Formatted: Font: (Default) Times New Roman, 12 pt

Formatted: Font: (Default) Times New Roman, 12 pt

Formatted: Font: (Default) Times New Roman, 12 pt

Formatted: Font: (Default) Times New Roman, 12 pt

Formatted: Left, Space After: 0 pt, Line spacing: single, Don't adjust space between Latin and Asian text, Don't adjust space between Asian text and

Ethics Committee of the University of Health Sciences, Gabon under the number 007/USS/18. All women admitted to the programme were admitted after obtaining their written informed consent.

Results

The average age of the women screened was 39.8 ± (SD =11.4) years with extremes of 25 and 83 years. Women aged 25-34 were the most represented, in the order of 40.5% of the sample. There were 625 women with a history of sexually transmitted infections, 41.3%. Of these, 534 had Chlamydia infection, 35 had HIV and 56 had gonorrhoea and syphilis. Smoking women accounted for 5.8% of the participants. Among the participants screened, 67.8% did not use a contraceptive method. The other characteristics of women are presented in Table 1.

Visual inspection and colposcopy results

In this study, 12.0% of women had a pathological cervix on unprepared examination. Women who tested positive for VIA and VIL accounted for 5.9% and 12.6% respectively. Colposcopy was only for cases with positive VIL, 191 tests or 12.6% (191/1512). With colposcopy, 46.1% of women had atypical grade1 transformations, 49.7% grade 2 and 4.2% had both atypical transformations (Table 2).

Histological results

In this study, of 63 women diagnosed histologically, 43 had precancerous lesions with 23 CIN I or 36.5%; 11 CIN II or 17.5% and 9 CIN III or 14.3%. Squamous cell cancers accounted for 20.6% and adenocarcinomas 3.2% (Table 3).

Risk factors of precancerous and cancerous lesions

Tables 4 and 5 show the characteristics that were risk factors for precancerous and cancerous lesions using bivariate and multivariable analysis. Using binary logistic regression to assess the relationship between cervical precancerous lesions and the independent variables collected, we found that being older than 35, having the first intercourse before 18, having an antecedent of STI, being a widow and ~~using~~ use of tobacco were risk factors associated with precancerous lesions (p = 0.013 with OR=3.44 (1.22-9.73), p = 0.009 with OR = 4.07 (1.69-13.08), p < 0.001 with OR = 3.80 (1.94 -7.47), p < 0.001 with OR = 9.77 (3.87-24.70) and p < 0.001 with OR = 5.47 (2.60-11.52)) respectively. With multivariate regression, the risk factors of developing any precancerous lesion were the same but the OR decreased with increased ~~party~~ parity (p = 0.04 with OR=1.44 (1.09-2.21), p = 0.04 with OR = 2.01 (1.99-2.61), p = 0.006 with OR = 1.97 (1.68-2.89), p = 0.021 with OR = 1.99 (1.30-2.37) and p = 0.02 with OR = 2.29 (1.14-2.99)) respectively. The total number of pregnancies and parity were not associated with precancerous lesions and cervical cancer. But after adjustement, only being older than 45, being a widow and using tobacco were risk factors associated with cancerous lesions (p = 0.021 with OR = 2.01 (1.58-3.56), p = 0.02 with OR = 2.96 (2.10-3.87), p = 0.041 with OR = 1.98 (1.46-2.44)) respectively.

Discussion

This study provided an update on the prevalence of precancerous cervical lesions in the obstetrics and gynaecology department. The target population was sexually active women 25 years of age and older. Several countries have succeeded in preventing 91% of all invasive cervical cancers by implementing generalized cytological screening[14]. Finally, the authors could have screened as many women in order to increase the power of the study.

Socio-demographic and gyneco-obstetrical characteristics

In this study, the mean age was 39.8 ±11.4 years with extremes ranging from 25 to 83 years. Some authors have worked on the same subject as us. For these authors, the average age ranged from 33 - 40 years[7,9,10,14-17] and was close to ours. The age at which screening begins is the subject of much debate among cervical cancer screening professionals. At the global level, there is no consensus on the age at which screening should begin. At 21 years of age (or one year after the first reports) in the United States and Switzerland[2], 25 years in England, France and according to WHO[3,4]. Cervical cancer is caused by an oncogenic sexually transmitted HPV infection. Therefore, any woman who has already had sexual intercourse should be regularly screened. The majority of the study population were single and concubine women (67.7%), however 28.1% were married. These results are similar to those found by ~~of Edith~~ MPIGA et *al*[15] ~~who found 43% of single women~~. The age at which screening begins in Gabon is 25 years old. ~~As a result, 70% of premenopausal women were in the majority~~It was found that 80.4 % of participants wre premenopausal. Paradoxically, 67.8% of women were not using contraceptives. This translates into a small percentage of ~~nulligest and~~ nulliparous people. In Gabon, women have the perception that contraception makes them sterile.

Formatted: Font: Italic

Screening results

In this series, 91 women had a pathological cervix, or 2%. The VIA and VIL tests were positive at 5.9% and 12.6% respectively. These results vary in some authors from 1.9% to 10.8% for VIA[10,15,18-20] and from 4.8% to 5.8% for VIL[13,15]. This difference is explained by the fact that women have been sensitized with an important message : cervical cancer screening reduces the risk of disease and mortality. The cytohistological analysis determined the prevalence of precancerous lesions at 2.8%. This prevalence seems high because of the large number of women screened, compared to some authors[20]. The screening programme should

focus on raising awareness among women, which is generally very successful and has shown that countries that have implemented cervical cancer screening reduce the burden of this disease[21,22].

Risk factors

We found that 41.3% of the women screened reported having contracted a STI. In our data base most of them contracted chlamydia infection and gonococcal disease. This high percentage of chlamydia infection in our population is of concern ; because Roset Bahmanyar et *al* in 2012 has established that, only the history of sexually transmitted infections (STIs) is significantly associated with a risk of cervical injury[23]. Unprotected sexual activity is associated with an increased risk of contracting STIs, leading to cervical inflammation which can promote the development of cervical dysplasia Unprotected sexual activity is associated with an increased risk of contracting STIs whose action on cervical inflammation and local immunity will promote the development of cervical dysplasia[24]. In this study 5.8% of the participants were smokers. Smoking seems to be strongly associated with the development of precancerous cervical lesions and cancer. Smoking is one of the most commonly identified environmental co-factors that can affect the risk of cervical cancer. The majority of women screened, 53.1% had their first sexual intercourse between 12-17 years of age. This result marks the precocity of sexual intercourse, which is a factor that favours precancerous lesions of the cervix[15,17,25]. It is important to continue with raising awareness among women about screening. Compared to married women, widows are more prone to develop precancerous and cancerous cervical lesions. We could not explore the reasons for widowhood, but many of these widows are on antiretroviral treatment. These results confirm the interest of organized cervical cancer screening in Gabon. The countries that have implemented this screening programme have made a significant contribution to reducing the burden of this disease[22].

Formatted: Font: Italic

This study had several limitations. As the study was a convenience sample of women presenting for care from only Libreville in Gabon, the prevalence results may not be generalizable to the whole of Gabon or to the Central African female population, where HIV infection rates are quite varied. There is also a possibility of selection bias, as women who participated in the screening program may have been more likely to have current symptoms. The main purpose of this study was to serve as a baseline for further studies and to help in planning for cervical cancer screening in Gabon ; future studies using randomized sampling techniques may give a more complete estimation of the national prevalence. Lastly, there were limited variables available for analysis in the medical records, limiting the number of risk factors and confounders that we were able to study, for exemple the antecedent of cancer family.

Conclusion

In this study, the participants in the cervical cancer screening at the UHCL in 2018 had an average age of 39 years, they were mostly single, premenopausal, multi-gestured. Early sexual intercourse and smokers were found. Among participants diagnosed with cervical cancer, there was a significant association with age, STI, marital status and smoking. Despite the integration of the detection of precancerous cervical cancer lesions into health facilities in Gabon, cervical cancer ranks second among women's cancers in terms of incidence and first in terms of mortality. These results help to further strengthen the fight against cervical cancer, provide clinicians with the characteristics of women with cervical cancer and allow further research to identify the factors that contribute to it. Thus, the detection and management of precancerous cervical lesions could reduce the mortality rate from cervical cancer in the long term.

Fundings : None

Conflicts of interest : The authors declare no conflict of interest

Authors' contributions :

NAL designed the study, wrote the protocol and text and supervised the data analysis. SHW, ASM and FEYM participated in the drafting of the protocol, data analysis and text writing. All authors have read and approved the manuscript and contributed equally.

Acknowledgements :

The authors would like to thank the Ministry of Health of Gabon

~~**Ethical approval**~~

~~University of Health Sciences, Gabon approved this study~~

Declarations : Availability of data and materials

The datasets used and analysed during the current study available from the corresponding author on reasonable request. The datasets generated and/or analysed during the current study are not publicly available due to the promise made to women to keep the data confidential when they are questioned, but are available from the corresponding author on reasonable request.

References

1. Bray F, Ferlay J, Soerjomataram I, Siegel RL, Torre LA, Jemal A. Global cancer statistics 2018: GLOBOCAN estimates of incidence and mortality worldwide for 36 cancers in 185 countries. CA Cancer J Clin 2018 ; 68 : 394-424 doi: 10.3322/caac.21492
2. Fitzmaurice C et al. The Global Burden of Cancer 2013. JAMA Oncol 2015 ; 1 : 505-27 doi: 10.1001/jamaoncol.2015.0735
3. Cohen PA, Jhingran A, Oaknin A, Denny L. Cervical Cancer. Lancet 2019 ; 3 : 169-182 doi: 10.1016/S0140-6736(18)32470-X.

4. Denny L, Anorlu R. Cervical cancer in Africa. Cancer Epidemiol Biomarkers Prev 2012 ; 21 : 1434-8 DOI: 10.1158/1055-9965.EPI-12-0334
5. Denny L. Cervical cancer :prevention and treatment. Discov Med 2012 ; 14 : 125-31
6. Comprehensive cervical cancer control : a guide to essential practice – 2nd ed. Geneva : World Health Organization ; 2017. Licence : CC BY-NC-SA 3.0 IGO
7. Denny L, Kuhn L, Pollack A, Wright TC Jr. Direct visual inspection for cervical cancer screening : an analysis of factors influencing test performance. Cancer 2002 ; 94 : 1699-707 DOI :10.1002/cncr.10381
8. Elfström KM, Arnheim-Dahlström L, von Karsa L, Dillner J. Cervical cancer screening in Europe : Quality assurance and organisation of programmes. Eur J Cancer 2015 ; 51 : 950-68
9. Saslow D, Solomon D, Lawson HW, Killackey M, Kulasingam SL, Cain J *et al*. American Cancer Society, American Society for Colposcopy and Cervical Pathology, and American Society for Clinical Pathology screening guidelines for the prevention and early detection of cervical cancer. CA Cancer J Clin 2012 ; 62 : 147-72
10. Sandjong Tietchou I, Sando Z, Tebeu PM, Mouelle Sone A, Essame Oyono JL, Doh Anderson S. Evaluation of the prevention and treatment of the precancerous lesions of the uterine cervix according to the 'see and treat' approach. Health Sci Dis 2015 ; 16
11. National Agency for Health Accreditation and Evaluation. Conduct in presence of an abnormal cervical smear. J Gynecol Obstet Biol Reprod 1999 ; 28 :310-18
12. Hasanzadeh M, Esmaeili H, Tabaee S, Samadi F. Evaluation of visual inspection with acetic acid as a feasible screening test for cervical neoplasia. J Obstet Gynaecol Res 2011 ; 37 :1802-6 doi: 10.1111/j.1447-0756.2011.01614.x.
13. Santesso N, Mustafa RA, Schünemann HJ, Arbyn M, Blumenthal PD, Cain J *et al*. World Health Organization Guidelines for treatment of cervical intraepithelial neoplasia 2-3 and

screen-and-treat strategies to prevent cervical cancer. Int J Gynaecol Obstet 2016 ; 132 : 252-8 doi: 10.1016/j.ijgo.2015.07.038

14. Denny L, Herrero R, Levin C *et al*. Cervical Cancer. In: Gelband H, Jha P, Sankaranarayanan R, et al., editors. Cancer : Disease Control Priorities, Third Edition (Volume 3). Washington (DC): The International Bank for Reconstruction and Development / The World Bank; 2015 Nov 1. Chapter 4. Available from: https://www.ncbi.nlm.nih.gov/books/NBK343648/ doi: 10.1596/978-1-4648-0349-9_ch4
15. Mpiga E, Ivanga M, Koumakpayi IH, Engohan-Aloghe C, Ankély JC, Belembaogo E *et al*. Interest in visual inspection with acetic acid and Lugol iodine with colposcope in screening of cervical lesions in Gabon. Pan Afr Med J 2015 ; 22 : 165 doi: 10.11604/pamj.2015.22.165.7038
16. Millogo FT, Akotionga M, Lankoande J. Cervix cancer screening in a health district (Burkina Faso) by biopsies after the application of acetic acid and lugol. Bull Soc Pathol Exot 2004 ; 97 : 135-38
17. Salifou K, Brun L, Akpona LFJ, Obossou AAA, Perrin RX. Factors associated with precancerous and cancerous cervical lesions in the city of Parakou, Benin. Eur Sci J 2015 ; 11 : 275-83
18. Phongsavan K, Phengsavanh A, Wahlström R, Marions L. Safety, feasibility, and acceptability of visual inspection with acetic acid and immediate treatment with cryotherapy in rural Laos. Int J Gynaecol Obstet 2011 ; 114 :268-72 doi: 10.1016/j.ijgo.2011.03.009.
19. Fallala MS, Mash R. Cervical cancer screening : Safety, acceptability, and feasibility of a single-visit approach in Bulawayo, Zimbabwe. Afr J Prim Health Care Fam Med 2015 ; 7 : doi: 10.4102/phcfm.v7i1.742.

20. Ngoma T, Muwonge R, Mwaiselage J, Kawegere J, Bukori P, Sankaranarayanan R. Evaluation of cervical visual inspection screening in Dar es Salaam, Tanzania. Int J Gynaecol Obstet 2010 ; 109 : 100-4 doi: 10.1016/j.ijgo.2009.11.025

21. Adefuye PO, Dada OA, Adefuye BO, Shorunmu TO, Akinyemi BO, Idowu-Ajiboye BO. Feasibility, acceptability, and effectiveness of visual inspection of the cervix with acetic acid and cryotherapy for dysplasia in Nigeria. Int J Gynaecol Obstet 2015 ; 129 : 62-6 doi: 10.1016/j.ijgo.2014.10.032

22. Denny L, Anorlu R. Cervical cancer in Africa. Cancer Epidemiol Biomarkers Prev 2012 ; 21 : 1434-8 DOI: 10.1158/1055-9965.EPI-12-0334

23. Roset Bahmanyar E, Paavonen J, Naud P, Salmerón J, Chow SN, Apter D *et al*. Prevalence and risk factors for cervical HPV infection and abnormalities in young adult women at enrolment in the multinational PATRICIA trial. Gynecol Oncol 2012 ; 127 :440-50 doi: 10.1016/j.ygyno.2012.08.033

24. Adefuye A, Sales K. Regulation of inflammatory pathways in cancer and infectious disease of the cervix. Scientifica (Cairo) 2012 ; 2012: 548150.PubMed/Google scholar

25. Makuza JD, Nsanzimana S, Muhimpundu MA, Pace LE, Ntaganira J, Riedel JR. Prevalence and risk factors for cervical cancer and precancerous lesions in Rwanda. Pan Afr Med J 2015 ; 22 : 26 doi:10.11604/pamj.2015.22.26.7116

Table 1. Sociodemographic and medical characteristics of participants

Variables		Number	Percentage
Age (years)			
	25-34	613	40.5
	35-44	427	28.2
	45-54	292	19.3
	55-64	141	9.3
	65 +	39	2.6
Marital status			
	Married	425	28.1
	Concubine/single	1024	67.7
	Widow	63	4.2
Hormonal status			

Formatted: Font: (Default) Times New Roman, 12 pt

Formatted: Normal, Indent: Left: 0"

	Postmenopausal	297	19.6
	Premenopausal	1215	80.4
Current contraceptive use			
	Yes	487	32.2
	No	1025	67.8
Gesture			
	Nulligest	155	10.3
	Primigest	154	10.2
	Multigest	1203	79.5
Parity			
	Nulliparous	206	13.6
	Primiparous	308	20·4
	Multiparous	998	66·0
First intercourse (years)			
	< 12	41	2.7
	12-17	802	53.1
	18-29	669	44.2
STIs			
	Yes	625	41.3
	No	887	58.7
Tobacco			
	Yes	87	5.8
	No	1425	94.2

Table 2. Distribution of women according to different types of tests

Visual inspection	N		Number	Percentage
Unprepared test	1512			
		Normal	1327	88.0
		Pathological	185	12.0
VIA test	1512			
		Negative	1423	94.1

		Positive	89	5.9
VIL test	1512			
		Negative	1321	87.4
		Positive	191	12.6
Colposcopy*	191			
		ATG 1	88	46.1
		ATG 2	95	49.7
		ATG 1 & ATG 2	08	4.2

* Positive cases only from VIL test

Table 3. Results from histological examinations for screened women

	Histology	Number	Percentage
Precancerous lesions			
	CIN 1	23	36.5
	CIN 2	11	17.5
	CIN 3	9	14.3
Cervical cancers			
	Squamous cell cancers	13	20.6

	Adenocarcinomas	07~~2~~	~~3.2~~11.1
	~~Other types of cancer~~	~~05~~	~~7.9~~
Total		63	100.0

Table 4. Risk factors of precancerous lesions

Risk factors	Histology			p	AOR* (95% CI)	p
	Injury	No injury	OR (95% CI)			
Age (years)						

25-34	05	608		1.0	1.0	-
35-44	25	402	7.56 (2.87-19.91)	< 0.001	2.81 (2.09-3.11)	0.05
45 +	13	459	3.44 (1.22-9.73)	0.013	1.44 (1.09-2.21)	0.04
Age on first intercourse						
< 12	05	36	6.06 (2.08-17.59)	0.004	1.66 (1.11 – 3.36)	0.03
12-17	23	779	4.07 (1.69-13.08)	0.009	2.01 (1.99-2.61)	0.04
18-29	15	654	1.0	-	1.0	
STIs						
Yes	31	594	3.80 (1.94 -7.47)	< 0.001	1.97 (1.68-2.89)	0.006
No	12	875	1.0	-	1.0	-
Marital status						
Married	09	416	1.0	-		
Concubine/Single	23	1021	1.04 (0.48-2.27)	0.55		
Widow	11	52	9.77 (3.87-24.70)	< 0.001	1.99 (1.30-2.37)	0.021
Tobacco						
Yes	10	77	5.47 (2.60-11.52)	< 0.001	2.29 (1.14-2.99)	0.02
No	33	1392	1.0		1.0	

* : Adjusted Odds ratio. Adjustment on gesture, parity and use of oral contraceptive

Table 5 : Risk factors for cancerous lesions

**Histology**

Risk factors	Injury	No injury	OR (95% CI)	p	AOR* (95% CI)	p
Age (years)						
25-34	3	610	1.0		1.0	
35-44	6	421	2.89 (0.72-11.65)	0.11	-	
45 +	11	461	4.85 (1.35-17.49)	0.008	2.01 (1.58-3.56)	0.021
Age on first intercourse						
< 12	4	37	10.22 (2.86-36.49)	0.0023	1.88 (0.97 – 2.01)	0.087
12-17	9	793	1.17 (0.40-2.89)	0.547		
18-29	7	662	1.0	-		
STIs						
Yes	14	611	3.36 (1.29 -8.80)	0.008	1.97 (1.68-2.89)	0.050
No	06	881	1.0	-	1.0	-
Marital status						
Married	04	421	1.0	-		
Concubine/Single	07	1017	0.72 (0.21-2.49)	0.410		
Widow	09	63	15.03 (4.49-50.28)	< 0.001	2.96 (2.10-3.87)	0.025
Tobacco						
Yes	05	82	5.73 (2.03-16.16)	0.004	1.98 (1.46-2.44)	0.041
No	15	1410	1.0		1.0	

* : Adjusted Odds ratio. Adjustment on gesture, parity and use of oral contraceptive
